# Gleanings from the German

**Published:** 1874-11-15

**Authors:** E. J. Doering


### GLEANINGS FROM THE GERMAN.

*Collated by Dr. E. J. Doering.*

### TREATMENT OF SPRAINS BY MASSAGE.

DR. FONTAINE is the author of the following article in the *Centralblatt fur Chirurgie*, No. 26, on the treatment of sprains by massage or shampooing.

For a number of years an old woman in the village of Thelin, in Belgium, has been celebrated for curing all sorts of sprains, acute as well as chronic cases—even such as resisted the skillful treatment of eminent practitioners—by massage, a process to be described presently. Prompted by her miraculous cures the author tried this method in a series of cases, and with the most brilliant results. In a comparatively short time he thus cured a rupture of the tendon of the m. plantaris, a sprain of the thumb and of the hand, and about a dozen cases of distorsio pedis, of which two were of a serious nature. The average duration of the treatment was from four to nine days in simple cases, and from fifteen to twenty days in severe cases. The efficiency of the treatment is well demonstrated by the following case;

An officer, by a fall from a horse, suffered a severe sprain of the tibio-tarsal articulation of the right foot, with considerable laceration of the ligaments and soft parts surrounding the outer malleolus; by massage the swelling subsided and the pain diminished so rapidly that the patient was enabled to walk about again in three days, and completely cured in two weeks.

Massage, or shampooing is performed in the following manner: First grease the affected extremity with lard, to protect the epidermis, then, with the thumb of the right hand, or with both thumbs, exert gentle pressure very slowly and steadily from below upwards, towards the heart, and always parallel to the direction of the tendons and muscular fibres. The friction over the affected painful part must be very gentle, but more and more energetic over the healthy portions of the limb, at times even kneading the muscles. This manipulation lasts from a quarter to half an hour, and must be repeated several times a day, according to the intensity of the sprain. Elevation of the foot and the application of a retaining bandage are of great service.

The author suggests the following as an explanation of the modus operandi of massage: The blood from the torn vessels, effused among the muscular fibres, aponeurotic fibres and tendinous sheaths, producing ecchymosis, and which is the cause of the great pain and swelling, is, by means of these frictions distributed over a larger surface, and therefore more rapidly absorbed, aided by the increased activity of the venous circulation from the direction of the frictions toward the heart. The author strongly condemns the practice of treating a sprain by applying an immovable bandage, as adhesions of the lacerated parts are apt to occur, causing stiffness of the joint and retarding a cure for months. Furthermore, other serious consequences of sprains, as acute and chronic inflammation of the joints, anchylosis, and especially tumor albus or white swelling, are nearly entirely obviated by massage. Another great advantage of this treatment is its simplicity, so that by a little instruction its proper performance can be safely intrusted to the nurse and relatives of the patient.

This treatment is not at all new, for as early as the year 1837 it was recommended by French physicians; of late, especially, by Phelippeaux (*Etude pratique sur les frictions et le massage, ou guide du medecine masseur.* Paris, 1870; and, *Contributions a la vulgarization du massage*). The author concludes with the emphatic words of Phelippeaux: "The universal introduction of massage into medical practice will prove to be one of the greatest boons ever conferred upon mankind."

### CAPILLARY EVACUATION OF CERVICAL ABSCESSES.

Dr. Crocq contributes to the *Allg. Med. Central Zeitung*, No. 79, the following article concerning his method of opening abscesses in the neck:

Although cervical adenitis is not classed among dangerous diseases, nevertheless it is the source of a great deal of trouble, especially on account of the subsequent disfigurement from scars. These cicatrices occur both when the abcess bursts spontaneously, or when it is opened artificially. In the male, fortunately, the beard generally conceals the scars from sight, but women are often thus disfigured for life. The physician therefore is frequently accused of carelessness in not having adopted the proper means for preventing scarring.

These facts induced the author to search for some method by which such abscesses might be rapidly and safely healed, and yet leave no unsightly disfigurement behind. If the abscess is allowed to break spontaneously the cavity heals very slowly and leaves an irregular scar. It is therefore the physician's duty to convince the patient that an artificial opening is far preferable.

But a cicatrix cannot be prevented from following an incision made by a bistoury, however small, but all these disadvantages may be obviated if the abscess is punctured by a very fine explorative trocar. If the abscess is large several punctures must be made to facilitate the flow of pus, which may be further aided by gentle pressure. The operation, which is entirely painless, must be repeated every second or third day. In a few days the pus diminishes in quantity and becomes serous, and the swelling subsides. After the abscess has healed small red points show where the punctures have been made, but these soon fade away, leaving after a little while no trace of the abscess. It is very desirable that the punctures should not be made in places where the skin is thinned, as suppuration is apt to result.

For the last ten years Dr. C. has employed this method, both in acute and chronic cases, as soon as fluctuation can be detected, and always with uniform success, for in no case has a cicatrix formed, or suppuration taken place.

Drs. Lawson Tait, in London, and Lorentzer, in Canstatt, have adopted a modification of the author's method, using Dieulafoy's aspirator, first puncturing the abscess with a hypodermic syringe. But both declare that the results obtained were not satisfactory. Dr. C. therefore concludes that his method is simpler, more efficacious, and not only applicable to abscesses of the lymphatic glands, but also to cutaneous abscesses following erysipelas and small-pox, several cases of which he has thus treated with the happiest results.

TREATMENT OF GONORRHŒA.

The following is an extract from a lengthy article by Dr. Haberkorn, in the *Berl. Klin. Wochenschrift*, No. 34, on the above subject:

Injections of permanganate of potassa, carbolic acid, sulphate of zinc, and other remedies, have all proved more or less insufficient in the treatment of gonorrhœa. After repeated experiments the author has found the sulphate of quinine to be a far superior remedy, being prompt in its action and nearly painless. He directs about a teaspoonful of the following mixture to be injected three times a day, retaining it for some time in the urethra:

℞ Quiniæ sulphat., gr. xv.

Acid. sulphur., dil. ℈j.

Glycerinæ, f ʒ vj.

Aquæ, f ℥ ij.

After three days a great improvement took place in all his cases. The expense of the medicine is covered by the rapidity of the cure. These results therefore justify a more extensive trial of this remedy.

TINCTURE EUCALYPTUS GLOBULES IN INTERMITTENT FEVER.

The following results are summed up by Dr. Hirsch, (*Berl. Klin. Wochenschrift*, No. 30) as obtained from his experiments with the tincture in nine cases of obstinate intermittent fever:

1. In all the cases, after the use of the remedy for one or more days, the spleen diminished in size.

2. In six cases, three, at most four, teaspoonfuls of the medicine were sufficient to prevent a return of the paroxysms. In one case only was the double quantity required.

3. Seven of the nine cases were cured completely; in the remaining two the remedy proved unsuccessful.

From these results Dr. H. draws the conclusion that tinct. eucalypt. glob. is a remedy but little, if any, inferior to quinine in the treatment of intermittent fever, and that it will probably prove to be as valuable an antiphlogistic in the treatment of other fevers as quinine, digitalis and veratrum.

